# Healthcare Provider Confidence and Perceived Barriers to Diagnosing Illness Associated with Harmful Algal Blooms

**DOI:** 10.1016/j.focus.2023.100154

**Published:** 2023-10-05

**Authors:** Marissa K. Vigar, Muhammad Thuneibat, Amy L. Jacobi, Ashley Andújar, Virginia A. Roberts

**Affiliations:** 1 National Center for Emerging and Zoonotic Infectious Diseases, Centers for Disease Control and Prevention, Atlanta, Georgia; 2 Oak Ridge Associated Universities, Oak Ridge, Tennessee

**Keywords:** Health communications, harmful algal blooms, healthcare providers, illness diagnostics

## Abstract

**Introduction:**

Illnesses caused by harmful algal blooms can vary in clinical presentation and severity depending on the types of algae, cyanobacteria, or toxins involved as well as the route and extent of exposure. These illnesses are primarily diagnoses of exclusion owing to lack of access to clinical testing for harmful algal bloom toxins. We used a national survey of healthcare providers to explore self-reported confidence and perceived barriers to diagnosing harmful algal bloom–associated illnesses.

**Methods:**

We conducted a descriptive analysis (SAS 9.4) of 2 questions from a national survey of healthcare providers administered September 14–October 26, 2020 (N=1,503). Chi-square tests were used to assess differences between groups, with statistical significance defined as *p*<0.05.

**Results:**

Most (68%) providers reported little to no confidence in their ability to identify illnesses caused by harmful algal blooms, 17% were confident or very confident, and 15% reported not seeing patients with these illnesses. Responses differed by specialization, work setting, sex, experience, whether pediatric patients were seen, and number of patients seen. Respondents regularly using resources such as newspapers (31%), professional societies (29%), and government health agencies (26%) reported increased confidence. Of 1,283 (85%) respondents who were asked about barriers to accurately diagnosing patients with these illnesses, 71% selected lack of knowledge about harmful algal bloom–associated illnesses as a barrier.

**Conclusions:**

Provider confidence in identifying harmful algal bloom–associated illnesses was low, and lack of knowledge was identified as a primary barrier, suggesting that more outreach and education are needed. Tailoring information for specific provider audiences and providing it through highly frequented channels could increase providers’ confidence in identifying and diagnosing harmful algal bloom–associated illnesses.

## Highlights

• Illnesses caused by harmful algal blooms are difficult to diagnose.
• A national survey of healthcare providers examined confidence and barriers to diagnosis.
• Few respondents were confident in their ability to diagnose these illnesses.
• Respondents cited a lack of knowledge as the most common barrier.
• Providing information through preferred channels could increase provider confidence.

## INTRODUCTION

Harmful algal blooms (HABs) occur when algae or cyanobacteria grow excessively, causing harm to humans, animals, or the environment. Factors that contribute to HABs in salt, brackish, and freshwater bodies include nutrient pollution (e.g., nitrogen and phosphorus run off from land-based sources) and warmer water temperatures.^1^ Climate change effects might alter the occurrence and severity of HABs in the U.S., increasing the risk to human health and well-being.^2^ Health effects in humans are usually associated with exposures to toxins produced during a HAB. Illnesses caused by HABs encompass a range of symptoms and severity, dependent on factors such as types and concentrations of algae, cyanobacteria, and toxins involved as well as exposure routes and pre-existing conditions (e.g., asthma).^3^, ^4^, ^5^, ^6^

To diagnose HAB-associated illnesses, providers need a basic awareness of HABs and the ability to identify clinical presentations and exposures. HAB-associated illnesses are primarily a diagnosis of exclusion because clinical testing options for HAB toxins are lacking; ideally, providers have access to clinical diagnostic testing to rule out other possible causes. Healthcare providers contribute to public health efforts when they identify and report HAB-associated illnesses to their jurisdictional health authority. For example, a healthcare provider diagnosis is a pathway to a confirmed case classification in the One Health Harmful Algal Bloom System, a national public health surveillance system used by state and territorial health departments.^7^ Electronic health records are being examined as another data source to characterize the human health impact of HABs.^8^ To explore the current state of confidence in identifying HAB-associated illnesses and perceived diagnostic barriers, we utilized a national survey of healthcare providers.

## METHODS

### Study Sample

We conducted a descriptive analysis (SAS, Version 9.4) of data from the 2020 Porter Novelli DocStyles survey, a national web-based survey examining healthcare provider attitudes and behaviors (https://styles.porternovelli.com/docstyles/). All respondents were enrolled in an opt-in panel of healthcare providers and paid an honorarium of $20–$100 for completing the survey. Response quotas were set on the basis of specialty (e.g., 1,000 primary care physicians, 250 per specialty group). Eligible respondents were those (1) actively practicing and seeing patients in the U.S.; (2) working in an individual, group, or hospital practice; and (3) who had been practicing for at least 3 years. Centers for Disease Control and Prevention (CDC) licensed 2 questions within the survey to assess provider confidence and barriers related to diagnosing HAB-associated illness in patients. Demographic and occupational characteristic data were also examined.

### Measures

Providers were first presented with a description of HABs and then asked about confidence: *Algae or cyanobacteria can form HABs in waterbodies and sometimes produce toxins. Common exposures are water, including aerosols, and consuming seafood. How confident are you in your ability to identify signs and symptoms of illness caused by HABs? Select one*. Responses were as follows: *Very confident (almost every time), Confident (most of the time), Somewhat confident (some of the time), Not confident (very rarely if at all)*, or *I do not see patients with these illnesses*.

Providers were then asked about perceived barriers: *What are the barriers, if any, to accurately diagnosing your patients with illness caused by HABs? Select all that apply*. Respondents could select from *Knowledge about these illnesses, Resources about clinical presentation, Access to diagnostic testing, Determining if my patients were exposed*, or *None of the above*.

### Statistical Analysis

The survey was administered between September 14 and October 26, 2020. A total of 1,503 providers, including primary care physicians, pediatricians, and nurse practitioners/physician assistants, completed the survey questions. The data set was deidentified before receipt by CDC; the analysis was exempt from CDC IRB review (this activity was deemed not to be research as defined in 45 CFR 46.102[l], and IRB review was not required). Chi-square tests were used to assess differences between groups, with statistical significance defined as *p*<0.05.

## RESULTS

Of the 1,503 respondents, most (68%) reported little to no confidence in their ability to identify signs and symptoms of illness caused by HABs, 17% were either confident or very confident, and 15% reported not seeing patients with HAB-associated illnesses. Responses differed significantly by doctor specialization, work setting, sex, practice years, number of patients seen per week, whether pediatric patients were seen, and number of pediatric patients seen per week (Table 1).

**Table 1:** Provider Confidence in Ability to Identify Signs and Symptoms of Illness Caused by HABs (N=1,503)

Characteristic	Not confident or somewhat confident *n* (%)	Confident or very confident *n* (%)	I do not see patients with these illnesses *n* (%)	Total *n* (%)
Total	1,028 (68)	255 (17)	220 (15)	1,503 (100)
**Doctor specialization**				
Family	312 (71)	81 (18)	48 (11)	441 (29)
Internist	357 (64)	116 (21)	86 (15)	559 (37)
Pediatrician	187 (74)	31 (12)	34 (13)	252 (17)
Nurse practitioner	92 (69)	13 (10)	29 (22)	134 (9)
Physician assistant	80 (68)	14 (12)	23 (20)	117 (8)
**Work setting**				
Individual outpatient practice	146 (59)	64 (26)	37 (15)	247 (16)
Group outpatient practice	736 (70)	158 (15)	153 (15)	1047 (70)
Inpatient practice	146 (70)	33 (16)	30 (14)	209 (14)
**Age, years**				
25–34	152 (70)	26 (12)	38 (18)	216 (14)
35–44	338 (72)	67 (14)	66 (14)	471 (31)
45–54	297 (68)	87 (20)	54 (12)	438 (29)
55–64	181 (64)	55 (20)	46 (16)	282 (19)
65–74	54 (61)	20 (23)	14 (16)	88 (6)
≥75	6 (75)	0 (0)	2 (25)	8 (1)
**Sex**				
Male	607 (67)	189 (21)	109 (12)	905 (60)
Female	421 (70)	66 (11)	111 (19)	598 (40)
**Practice years**				
3–9	316 (73)	45 (10)	74 (17)	435 (29)
10–20	372 (68)	109 (20)	70 (13)	551 (37)
20–30	242 (66)	73 (20)	52 (14)	367 (24)
30–40	92 (65)	26 (18)	23 (16)	141 (9)
≥40	6 (67)	2 (22)	1 (11)	9 (1)
**Number of patients**^a^				
10–59	175 (71)	19 (8)	52 (21)	246 (16)
60–79	181 (71)	28 (11)	45 (18)	254 (17)
80–99	153 (73)	29 (14)	27 (13)	209 (14)
100–124	327 (72)	78 (17)	51 (11)	456 (30)
≥125	192 (57)	101 (30)	45 (13)	338 (22)
**Sees pediatric patients**^b^				
Yes	734 (69)	202 (19)	129 (12)	1065 (71)
No	294 (67)	53 (12)	91 (21)	438 (29)
**Number of pediatric patients**^a^**(*****n*****=1,065)**				
1–9	102 (76)	13 (10)	20 (15)	135 (13)
10–19	121 (71)	23 (13)	27 (16)	171 (16)
20–34	177 (69)	50 (19)	30 (12)	257 (17)
35–69	162 (65)	68 (27)	19 (8)	249 (23)
≥70	172 (68)	48 (19)	33 (13)	253 (24)
Region				
Midwest	208 (63)	72 (22)	49 (15)	329 (22)
Northeast	232 (69)	49 (15)	57 (17)	338 (22)
South	369 (72)	80 (16)	64 (12)	513 (34)
West	219 (68)	54 (17)	50 (15)	323 (21)
Metropolitan				
Urban	355 (65)	102 (19)	86 (16)	543 (36)
Suburban	540 (70)	121 (16)	116 (15)	777 (52)
Rural	133 (73)	32 (17)	18 (10)	183 (12)
**Uses government agencies such as CDC or NIH to keep up with medical news and trends**				
Regularly	159 (65)	63 (26)	22 (9)	244 (16)
Often	337 (68)	89 (18)	70 (14)	496 (33)
Sometimes	400 (71)	79 (14)	85 (15)	564 (38)
Rarely	111 (65)	23 (13)	37 (22)	171 (11)
Never	21 (75)	1 (4)	6 (21)	28 (2)
**Uses medical websites to keep up with medical news and trends**				
Regularly	354 (68)	90 (17)	75 (14)	519 (35)
Often	398 (69)	104 (18)	76 (13)	578 (38)
Sometimes	250 (70)	52 (15)	53 (15)	355 (24)
Rarely	24 (52)	9 (20)	13 (28)	46 (3)
Never	2 (40)	0 (0)	3 (60)	5 (0)
**Uses medical journals to keep up with medical news and trends**				
Regularly	295 (67)	105 (24)	42 (10)	442 (29)
Often	364 (68)	87 (16)	82 (15)	533 (35)
Sometimes	290 (70)	53 (13)	70 (17)	413 (27)
Rarely	73 (71)	9 (9)	21 (20)	103 (7)
Never	6 (50)	1 (8)	5 (42)	12 (1)
**Uses newspaper stories/articles to keep up with medical news and trends**				
Regularly	58 (56)	32 (31)	14 (13)	104 (7)
Often	185 (67)	62 (22)	29 (11)	276 (18)
Sometimes	365 (66)	102 (18)	85 (15)	552 (37)
Rarely	331 (76)	42 (10)	61 (14)	434 (29)
Never	89 (65)	17 (12)	31 (23)	137 (9)
**Uses professional medical societies to keep up with medical news and trends**				
Regularly	146 (60)	70 (29)	26 (11)	242 (16)
Often	347 (69)	90 (18)	66 (13)	503 (33)
Sometimes	392 (72)	78 (14)	77 (14)	547 (36)
Rarely	119 (70)	14 (8)	38 (22)	171 (11)
Never	24 (60)	3 (8)	13 (33)	40 (3)

*Note:* Boldface indicates statistical significance (*p*<0.05) between subcategories.

a Per week.

b Includes pediatric doctors. CDC, Centers for Disease Control and Prevention; HAB, harmful algal bloom.

Internists (21%) and family practitioners (18%) were more likely to be confident or very confident than other providers, and pediatricians were least confident (74%). Nurse practitioners (22%) and physician assistants (20%) were more likely to report not seeing patients with these illnesses. Confidence increased with provider age (23%), years in practice (22%), number of patients seen per week (30%), and number of pediatric patients seen per week (27%).

Respondents who regularly used newspapers (31%), professional medical societies (29%), government health agencies (26%), medical journals (24%), or medical websites (17%) to keep up to date with the latest medical news and trends reported increased confidence compared with those who used these sources less often (Table 1).

Of the 1,503 respondents, 1,283 (85%) reported seeing patients with these illnesses and were asked about barriers to accurately diagnosing HAB-associated illnesses. Most providers reported knowledge about illnesses as a barrier (71%), followed by access to diagnostic testing (48%), resources about clinical presentation (46%), and determining whether their patients were exposed (44%). Only 3% of providers selected none of the above barriers (Table 2).

**Table 2:** Perceived Barriers, If Any, to Accurately Diagnosing Patients With Illness Caused by HABs (*n*=1,283)

Characteristic	Knowledge about illnesses *n* (%)	Resources about clinical presentation *n* (%)	Access to diagnostic testing *n* (%)	Determining patient exposure *n* (%)	None of the above *n* (%)
Total	905 (71)	590 (46)	619 (48)	569 (44)	43 (3)
Doctor specialization					
Family practitioner	276 (70)	178 (45)	178 (45)	183 (46)	9 (2)
Internist	326 (68)	226 (47)	239 (50)	226 (47)	17 (3)
Pediatrician	159 (72)	94 (43)	114 (52)	83 (38)	6 (2)
Nurse practitioner	79 (75)	52 (49)	45 (42)	40 (38)	6 (5)
Physician assistant	65 (69)	40 (42)	43 (45)	37 (39)	5 (5)
Work setting					
Individual outpatient	**133 (63)**	**109 (51)**	95 (45)	90 (42)	8 (3)
Group outpatient	**646 (72)**	**411 (45)**	450 (50)	398 (44)	27 (3)
Inpatient	**126 (70)**	**70 (39)**	74 (41)	81 (45)	8 (4)
Age, years					
25–34	136 (76)	75 (42)	79 (44)	71 (39)	10 (5)
35–44	294 (72)	195 (48)	183 (45)	184 (45)	12 (2)
45–54	260 (67)	169 (44)	196 (51)	169 (44)	9 (2)
55–64	161 (68)	116 (49)	121 (51)	104 (44)	9 (3)
65–74	49 (66)	32 (43)	37 (50)	37 (50)	3 (4)
≥75	5 (83)	3 (50)	3 (50)	4 (66)	0 (0)
Sex					
Male	556 (69)	374 (46)	389 (48)	365 (45)	**19 (2)**
Female	349 (71)	216 (44)	230 (47)	204 (41)	**24 (4)**
Practice years					
3–9	**276 (76)**	161 (44)	166 (45)	155 (42)	12 (3)
10–20	**337 (70)**	224 (46)	227 (47)	212 (44)	18 (3)
20–30	**211 (66)**	150 (47)	164 (52)	137 (43)	10 (3)
30–40	**75 (63)**	54 (45)	0 (0)	61 (51)	3 (2)
Number of patients^a^					
10–59	**148 (76)**	84 (43)	**80 (41)**	80 (41)	6 (3)
60–79	**147 (70)**	95 (45)	**87 (41)**	93 (44)	10 (4)
80–99	**142 (78)**	91 (50)	**97 (53)**	86 (47)	5 (2)
100–124	**289 (71)**	194 (47)	**201 (49)**	175 (43)	8 (1)
≥125	**179 (61)**	126 (43)	**154 (52)**	135 (46)	14 (4)
Sees pediatric patients^b^					
Yes	650 (69)	429 (45)	459 (49)	416 (44)	29 (3)
No	255 (73)	161 (46)	160 (46)	153 (44)	14 (4)
Number of pediatric patients^b^					
1–9	**92 (80)**	63 (54)	**67 (58)**	56 (48)	1 (0)
10–19	**103 (71)**	62 (43)	**61 (42)**	64 (44)	6 (4)
20–34	**151 (66)**	99 (43)	**95 (41)**	109 (48)	11 (4)
35–69	**149 (64)**	114 (49)	**127 (55)**	106 (46)	5 (2)
≥70	**155 (70)**	91 (41)	**109 (49)**	81 (36)	6 (2)
Metropolitan					
Urban	313 (68)	207 (45)	229 (50)	211 (46)	15 (3)
Suburban	474 (71)	308 (46)	314 (47)	294 (44)	23 (3)
Rural	118 (71)	75 (45)	76 (46)	64 (38)	5 (3)
Provider confidence					.
Not confident/somewhat confident	**787 (77)**	478 (47)	486 (47)	442 (43)	**28 (3)**
Confident/very confident	**118 (46)**	112 (44)	133 (52)	127 (50)	**15 (6)**

*Note:* Boldface indicates statistical significance (*p*<0.05).

a Per week.

b Includes pediatric doctors. HAB, harmful algal bloom.

Knowledge responses differed significantly by work setting, practice years, number of patients and pediatric patients seen per week, and confidence in identifying signs and symptoms of illness. Providers with more years of practice were less likely to report knowledge as a barrier, particularly among those with ≥30 years of experience (63%). Lack of knowledge as a barrier was least reported by providers who saw ≥125 patients of any age (61%) and most reported by those who saw <10 pediatric patients (80%) per week. Providers with less confidence in sign or symptom identification were more likely to report a lack of knowledge than those with more confidence (46% vs 77%) (Table 2).

Diagnostic testing responses differed significantly by number of patients and pediatric patients per week; seeing ≥80 patients per week or the fewest (<10) or most (≥35) pediatric patients per week was associated with an increased need. Lack of resources as a barrier differed significantly by work setting; relative to providers of other work settings, individual outpatient providers identified a lack of resources about clinical presentation (51%) more frequently and a lack of knowledge (63%) about HAB-associated illnesses less frequently (Table 2).

## DISCUSSION

Most respondents (68%) reported little to no confidence in their ability to identify HAB-associated illnesses. Although confidence differed by factors such as specialty and years of practice, provider confidence was low (<30% confident or very confident) across all groups. Most respondents (71%) selected knowledge as a barrier to accurately diagnosing illnesses, and those who selected it were less likely to indicate additional barriers, suggesting that more knowledge is foundational to advancing providers’ ability to accurately diagnose patients. Providers who reported little or no confidence were also more likely to report knowledge about illnesses as a barrier. These findings, paired with reported barriers related to resources on clinical presentation and patient exposure, suggest that provider outreach could improve illness identification and diagnosis. Consideration of additional outreach to communicate which HAB-associated illnesses are of public health interest could further improve official reporting of these illnesses once diagnosed.

Illness in children accounted for 40% of HAB-associated cases in 2019 and 25% of cases in 2020 reported to CDC.^9^^,^^10^ These data suggest that children might be disproportionately impacted by HABs. Although seeing more patients per week, including pediatric patients, was correlated with increased confidence, pediatricians reported less confidence than any other occupational specialty. Therefore, pediatricians should be considered a key audience for information about HABs.

Using newspapers, professional medical societies, governmental agencies, medical journals, and medical websites as sources of information was positively correlated with provider confidence. Organizations conducting physician outreach should consider prioritizing these channels while exploring methods to reach additional providers.

### Limitations

These survey results represent respondents’ self-assessments, and we cannot quantify their accuracy. In addition, confidence is not equivalent to competence, a more difficult quality to assess. The survey did not distinguish between (1) providers not seeing patients with HAB-associated illnesses and those unaware of seeing them or (2) providers who did not perceive any barriers and those who did not find a relevant barrier listed. Finally, there may be unmeasured factors contributing to differences in the frequency with which providers are using the types of channels described earlier (e.g., governmental agencies, professional medical societies).

## CONCLUSIONS

Provider confidence in their ability to identify and accurately diagnose HAB-associated illnesses was low among all medical specialties, with knowledge about these illnesses identified as a primary barrier to confidence. Continued efforts to educate providers about HAB-associated illnesses, including how they occur and their clinical presentations, are needed to improve broader understanding and prevention of these illnesses nationally. Focused outreach should consider the potentially distinct needs of early career providers and pediatricians. This could leverage existing mechanisms for training and evaluating competency, such as through continuing medical education courses and medical examinations. Resource channels that are frequently used by providers can be leveraged to deliver information with the goal of further addressing knowledge gaps.

## CRediT authorship contribution statement

**Marissa K. Vigar:** Conceptualization, Formal analysis, Software, Writing – original draft. **Muhammad Thuneibat:** Formal analysis, Software, Validation. **Amy L. Jacobi:** Conceptualization, Writing – review & editing. **Ashley Andújar:** Conceptualization, Writing – review & editing. **Virginia A. Roberts:** Conceptualization, Writing – review & editing, Supervision.
